# A scrub typhus vaccine presents a challenging unmet need

**DOI:** 10.1038/s41541-023-00605-1

**Published:** 2023-02-09

**Authors:** David H. Walker, Nicole L. Mendell

**Affiliations:** Department of Pathology, The University of Texas Medical Branch at Galveston, 301 University Blvd., Galveston, TX 77555 USA

**Keywords:** Infectious diseases, Infection

## Abstract

Scrub typhus caused by the obligately intracellular bacterium, *Orientia tsutsugamushi*, is a major cause of life-threatening acute undifferentiated febrile illness in eastern Asia and the islands of the Western Pacific and Indian oceans. Since the estimation of an incidence of 1 million cases annually two decades ago, the number of cases has increased substantially in endemic regions, reappeared where the disease was forgotten, and spread northward. Trombiculid mites are both reservoir and vector. Despite 80 years of efforts to develop a vaccine, there is none. Protective immunity is mediated by antibodies and CD8 and CD4 T cells. Previous efforts have failed because of gaps in understanding immunity to *O. tsutsugamushi*, particularly the requirements for vaccine-induced immunity, lack of knowledge regarding immune memory in scrub typhus, and lack of attention to addressing the issue of cross-protection between strains. There are numerous strains of *O. tsutsugamushi*, and modestly durable immunity is strain-specific. Antibodies to the strain that caused infection are protective against challenges with the homologous strain but, despite reactivity with other immunodominant antigens, the immune serum does not protect against heterologous strains. Among the antigens detected by western immunoblot in immune sera (22-, 47-, 56-, 58-, and 110 kDa proteins), only the 56 kDa protein stimulates strong protection. This protein contains four hypervariable regions which are likely, on the basis of limited data, to be the targets of neutralizing antibodies. However, a method that definitively detects neutralizing antibody has yet to be developed. Only one study has used genomic data to pursue the discovery of protective antigens. Three conserved autotransporters were identified, and only immunization with ScaA provided protection against the homologous strain, but only 40% of animals were protected against challenge with a heterologous strain. A multiplex vaccine containing conformational antigens of the hypervariable regions of the 56 kDa protein of the strains of the greatest clinical and epidemiological importance, as well as conserved regions of the 56 kDa protein, ScaA, and other protective antigens identified by future genomic and bioinformatics methods should be developed and tested.

## Introduction

Scrub typhus caused by *Orientia tsutsugamushi* is a major cause of life-threatening acute undifferentiated febrile illness in eastern Asia and the islands of the Western Pacific and Indian oceans^1^. The disease is also present in northern Australia, and illnesses caused by recently identified organisms in the genus *Orientia* are emerging in South America and Africa^2–5^. It has been stated two decades ago that the incidence of scrub typhus was 1 million cases annually. During the intervening period, the incidence has increased substantially in regions where the disease is recognized as endemic including China, Korea, Taiwan, and Thailand, has reappeared in regions where it had been forgotten, including enormous numbers of cases in India, and has spread northward of previously established endemic areas such as in China and Korea^6–10^.

*Orientia tsutsugamushi* is maintained in nature by transovarial transmission in trombiculid mites, which serve as both reservoir and vector^1^. The infected eggs hatch and infected chiggers, emerge from the soil to obtain a tissue fluid meal by attaching to the skin of a rodent as well as humans, both of which are dead-end hosts. During feeding on human skin, they inoculate *O. tsutsugamushi* in their salivary secretions. Orientiae infects primarily dendritic cells in the skin where a 1 cm area of dermal and epidermal necrosis leading to a crust or ulcer, the eschar, appears prior to the onset of systemic symptoms. Orientiae spread via lymphatic vessels to the regional lymph nodes that become enlarged, and the organisms are disseminated hematogenously infecting primarily endothelial cells throughout the body. Illness typically manifests with symptoms that often include fever, headache, myalgia, generalized lymphadenopathy, transient hearing loss, and rash. Untreated infection can progress with the development of acute respiratory distress, meningoencephalitis, acute renal failure, gastrointestinal bleeding, coagulopathy, and hypotensive shock. The untreated case fatality rate is 6%, and if treated 1.4%^11^. There appears to be variation in severity with much higher fatality rates reported in some outbreaks. There are very limited data that there may be strain virulence differences contributing to a range of disease outcomes^12^.

Despite attempts to develop a vaccine during the past 80 years, an effective vaccine has not been produced^13^. Approaches to vaccine development have included formalin-fixed homogenized lungs from *O. tsutsugamushi*-infected cotton rats^14,15^, other sources of formalin-killed *O. tsutsugamushi*^16–19^, a live low-virulence strain of *O. tsutsugamushi*, inoculation of live virulent organisms followed by chloramphenicol treatment^20,21^, live organisms treated with tetracycline^22,23^, and live irradiated *O. tsutsugamushi*^24–26^.

## Contribution of immune components to controlling *Orientia tsutsugamushi* infection

Investigations of immunity to *O. tsutsugamushi* include both studies of the components of the immune system that mediate recovery from an acute infection of experimental animals and studies of immune components that when present prior to infection prevent or significantly ameliorate experimental infection, such as would be stimulated by an effective vaccine. Our understanding of the immune components critical to controlling primary infection has been established through studies employing knockout mice. Animals genetically deficient in CD8 T cells (CD8, beta 2 microglobulin, and MHCI knockout mice) die with increased bacterial loads when challenged with an ordinarily sublethal dose of *O. tsutsugamushi*^27,28^. Observation of no difference in the outcome of infection between CD1d knockout mice and wild type mice suggests that NKT cells do not play a critical role in immunity to acute *O. tsutsugamushi* infection^28^. The roles of humoral immunity and CD4 T cells during primary infection have not been investigated in vivo.

A critical issue for vaccine development is the identification of the components of the adaptive immune system that must be stimulated by a vaccine to successfully prevent illness. Only limited studies of the role of particular immune components passively transferred prior to challenge with *O*. *tsutsugamushi* have been performed. Protection is conferred by the adoptive transfer of immune CD8 T cells from mice that have recovered from *O. tsutsugamushi* infection^27,28^. Similarly adoptive transfer of a CD4 T cell line that produced gamma interferon protected strongly against challenge by the homologous Gilliam strain with which the T cell line was derived, protected partially against Karp strain, but did not protect against Kato strain^29^. These results suggest that CD4 T cell protection is strain-dependent. Further, the adoptive transfer of CD8 T cell-depleted immune cells conferred some protection against the homologous strain^27^. Pioneering research by Shirai et al. in 1976 had shown that adoptive transfer of splenic T cells, but not B cells, from mice infected with the Gilliam strain 14–28 days earlier were protective against Karp strain^30^. Unfortunately, this cross-protective immunity was short-lived. Significantly, antibody to *O. tsutsugamushi* is highly protective. Passive transfer of immune serum including polyclonal antibodies is protective against the *O. tsutsugamushi* strain that caused the original infection but is not protective against other strains^31,32^. It is important to recognize that while such polyclonal sera are reactive with the numerous strain-shared immunodominant antigens these antibodies are not protective against other strains. Conserved immunodominant antigens do not appear to play an important role in humoral immunity although evaluation of immunization with individual antigens could conceivably discover cross-protective subdominant antigens. Moreover, cross-protection between strains after infection is short-lived, and even protection against the homologous strain wanes after a few years.

## Antigenic domains important in scrub typhus

Much of the protective antigen discovery research on *O. tsutsugamushi* has been focused on antigens detected in western immunoblots, mainly the 22-, 47-, 56-, 58-, and 110 kDa proteins (Table 1)^33–38^. The 56 kDa major immunodominant surface protein (p56), which is involved in fibronectin binding and facilitates host cell invasion, stimulates protection against disease caused by the same *O. tsutsugamushi* strain^39–43^. The open reading frame of p56 encodes 516–541 amino acids and based on the amino acid variability between isolates has four hypervariable regions^44^. Numerous phylodendrograms of genetic relationships of *O. tsutsugamushi* isolates have been constructed based on p56 sequences placing organisms in clades named according to a prototype serotype of *O. tsutsugamushi* such as Karp, Kato, and Gilliam strains. Genetic differences in the hypervariable regions of p56 of different strains within a clade may encode antigens that are not cross-neutralized^39^. The relationship between genotype and serotype is unclear. Cross protection that is stimulated by the p56 of *O. tsutsugamushi* genotypes within a phylogenetic clade has not been determined.

**Table 1:** Summary of important *Orientia tsutsugamushi* antigenic domains.

Antigen	Function	Similarity among genomes	Evidence for homologous strain protection	Evidence for heterologous strain protection
22 kDa	Unknown	Promoter regions conserved, variation in ORFs^a^ ^58^	DNA vaccine not protective in CD-1 mice^50^	Not tested
47 kDa	High-temperature requirement A (HtrA) protein family: serine protease and chaperone activities	97.4 to 100% amino acid similarity of ORF^b^ ^59^	DNA plasmid and recombinant protein partially protective in mice^49,50^ DNA plasmid vaccine or DNA plasmid/ Virus-vectored vaccine regimen protected NHPs from bacteremia (25%), fever, or eschar^48^	Not demonstrated
56 kDa	Attachment/invasion through fibronectin interaction^43^	78.8–90.2% amino acid similarity^e^ ^51^	Recombinant Boryong strain protein protective, and recombinant Karp strain protein and plasmid DNA are partially protective in mice^50,51,60,61^ Recombinant protein is immunogenic in NHP^62^	Recombinant Boryong strain protein conferred dose-dependent protection against the Karp strain and partial protection against and Kato strain in mice^51^
58 kDa	Heat shock protein homolog^35^	Unique, noncrossreactive as well as common epitopes^c^ ^63,64^	Not tested	Not tested
110 kDa	Unknown	Strain-specific epitopes^c^ ^34^	Recombinant plasmid vaccine conferred partial protection CD-1 mice^50^	Not tested
*ScaA*	Autotransporter: Mediates adhesion via vMLL5 to nonphagocytic cells^51,57^	Passenger domains conserved^e^ 81.0–88.4% amino acid similarity ^d^ ^51^	Recombinant protein derived from Boryong strain protective in mice^51,52^	Recombinant protein derived from Boryong strain protective against Karp strain challenge and partially protective against Kato strain (40%) in mice^51^
*ScaB*	Autotransporter: Adhesion to and invasion of nonphagocytic cells^65^	No.^e^ Absent in Ikeda strain^53^	Not tested	Not tested
*ScaC*	Autotransporter: Adhesion to nonphagocytic cells through potential fibronectin interaction^53^	Passenger domains conserved 77.4–97.5% amino acid similarity^e^ ^53,66^	ScaC recombinant protein derived from Boryong strain not protective in mice^51^	Not tested
*ScaD*	Autotransporter	Variation in internal repeat sequences in passenger domain^66^ 69.7–93.8% amino acid similarity^e^ ^66^	Not tested	Not tested
*ScaE*	Autotransporter	No. Passenger domain not detected in Kato strain 62.8–85.3% amino acid similarity^e^ ^66^	Not tested	Not tested

^a^Kato, TA763, AFSC 7, 18-032460, TH1814, and MAK 119.

^b^Karp, Kato, Ikeda, Gilliam, Boryong, TA763, TH1811 and TH1817, and MAK243, and isolates from Thailand, Laos, and Australia.

^c^Karp, Kato, and Gilliam.

^d^Boryong, Karp, Kato, and Gilliam.

^e^Boryong, Ikeda, Karp, Kato, and Gilliam.

Approaches to identify antibodies and antigenic domains important in scrub typhus infection have focused on reactivity and have inadequately addressed pathogen neutralization and antigen conformation. Demonstration of reactivity of antibodies with p56 has utilized EIA and immunoblotting techniques that may not preserve important conformational epitopes. Two approaches have addressed the demonstration of neutralizing antibodies. In 1983 Barbara Hanson determined that attachment/entry of the Gilliam strain into cells could be prevented by incubating hyperimmune sera containing antibodies against the Gilliam strain with *Orientia* for 3–5 h as observed by counting organisms by microscopy after washing away nonadherent organisms^45^. Seong et al. used a similar approach to evaluate two monoclonal antibodies against antigenic p56 domain II of *O. tsutsugamushi* Boryong strain by incubation of monoclonal antibody-containing mouse ascites fluid with *O. tsutsugamushi* organisms and endothelial cell line ECV 304 for 3 h, washing three times, and counting cell-associated *O. tsutsugamushi* by immunofluorescence microscopy^39^. The monoclonal antibodies were shown by competition EIA to be directed against amino acids 140–160 and 187–214, respectively, of p56. The neutralizing monoclonal antibodies that provided protection were directed against variable domain II, which is predicted to be a hydrophilic antigenic region. No other studies of antigenic stimulation of protection have evaluated the presence of neutralizing antibodies in vitro. Antibody neutralization cannot be concluded from these experiments due to the limitations of these technical approaches which did not determine whether infectivity was neutralized. Another large study evaluated the reactivity of 40 strains of *O. tsutsugamushi* with 15 monoclonal antibodies against p56^46^. Five of the six anti-Gilliam strain p56 monoclonals also reacted with seven other strains, which also reacted with an anti-Kawasaki strain monoclonal antibody. Two of the strains reacted with two anti-Karp strain p56 monoclonals. Five *O. tsutsugamushi* strains reacted with only the anti-Karp strain monoclonal. Kuroki strain and seven other strains reacted strongly with the anti-Kuroki strain p56 monoclonal as well as at a lower titer with an anti-Kawasaki strain monoclonal antibody. This study determined that there are epitopes in the *O. tsutsugamushi* p56 that are shared to a limited degree between some strains but do not reveal whether or not antibodies against any of the epitopes are protective. However, questions remain regarding which domains of the p56 monoclonal antibodies react, whether the epitopes are conformational, and the neutralizing capacity of the monoclonal antibodies.

Another approach to the identification of antigenic domains of *O. tsutsugamushi* p56 involved evaluating the reactivity of recombinant bacterial expressed fragments of p56 with sera of mice hyperimmunized with *O. tsutsugamushi* Karp, Gilliam, Kato, or Boryong strain^47^. The recombinant peptides containing variable domain I (VD I) of each strain reacted specifically with homologous sera from mice immunized with three of the four strains. In addition, the recombinant peptides containing VD II were reactive with strain-specific antibodies of all four strains, and there were crossreactions of recombinant peptides of Karp and Boryong strains of which the VD II peptides are 70% similar. None of the sera were reactive with the recombinant peptides containing VD III. Recombinant peptides containing VD IV were cross-reactive. In general, the reactive antigens of the recombinant peptides were likely linear, and it is unknown whether the peptide antigens contain conformational or non confirmational antigens that would stimulate neutralizing antibodies.

In another investigation competition, EIA was employed to determine the reactivity of patients’ sera, mouse monoclonal antibodies, and polyclonal mouse anti-recombinant p56 sera with recombinant peptides expressed by deletion constructs of *O. tsutsugamushi* Boryong strain^42^. Convalescent human IgG reacted with an antigenic domain I (amino acids 19–113) and antigenic domain III (amino acids 243–328), neither of which contains a variable domain. Mouse anti-recombinant p56 reacted with antigenic domain II (which contains VD II) and with antigenic domain III. These data do not elucidate which domains of p56 stimulate neutralizing antibodies and protective immunity nor whether the reactive antigens are conformational. It is logical that strain antigenic specificity would reside in the variable regions that contain diverse amino acid sequences. Identification of which variable domains of p56 in a series of the clinically and epidemiologically important *O. tsutsugamushi* strains stimulate neutralizing antibodies and protection against virulent challenges would be a valuable step toward the design and development of a future multiplex subunit vaccine. It should be determined whether the surface-exposed, likely hydrophilic antigens that are targets of neutralizing antibodies are conformational.

In order to determine whether regions of *O. tsutsugamushi* p56 contain antigens that would stimulate protective immunity, Kim et al designed a recombinant vaccine based on conserved sequences of 206 strains which consisted of seven conserved blocks of pairwise similarity of 47.5–76.9% that encoded amino acids that were greater than 77.6% conserved^41^. After three immunizations with the recombinant protein, mice were challenged intraperitoneally with 100 LD50 of *O. tsutsugamushi*. All mice became ill with 100% survival of animals infected with the Boryong and Karp strains and 40% survival of mice challenged with the Kato strain. This antigen merits consideration for inclusion in a multivalent subunit vaccine but would not be effective alone.

As presented above, p56 stimulates strong protection against homologous challenge but only weak protection against heterologous strains, and recombinant protein containing conserved regions of p56 provides protection against death, but not against illness, caused by Boryong and Karp strains but does not protect against Kato strain.

The 47 kDa (p47) antigen has been described as partially protective on the basis of reduction in eschars, delayed onset, and shortened course of illness in vaccinated nonhuman primates and absence of fever and bacteria in sampled blood in 25% of animals, but the great majority of the animals developed fever and bacteremia^48^. In a study of mice immunized intranasally three times with recombinant p47 and cholera toxin adjuvant, all animals became ill with disseminated infection with *O. tsutsugamushi* present in the heart, lung, liver, and spleen^49^. Other investigations at the Naval Medical Research Center that were reported as unpublished data stated that immunization with a 110 kDa DNA vaccine stimulated partial protection against homologous challenge without information on illness and death of the challenged animals or statistical analysis of protection and that a 22 kDa DNA vaccine was not protective^50^. The current evidence regarding the 22 kDa and 110 kDa proteins indicates that they do not merit inclusion in a multiplex scrub typhus vaccine.

Approaches to the development of a protective vaccine have taken little advantage of the availability of genome sequences of a growing number of *O. tsutsugamushi* strains. An exception has been the analysis of autotransporters. Three conserved autotransporters were identified in the Boryong, Ikeda, Karp, Kato, and Gilliam strain genomes, ScaA, ScaC, and ScaD. ScaB is absent from the Ikeda strain, and ScaE differs among the strains^51–53^. ScaA plays a role in the attachment of *O. tsutsugamushi* to the host cell, and antibody to ScaA neutralizes the attachment and entry of *O. tsutsugamushi* into the host cell. Immunization with ScaA of the Boryong strain protected all mice inoculated intraperitoneally with 100 LD50 of Boryong or Karp strain, but protected only 40% of mice challenged with Kato strain^51^. Although ScaC is involved in adhesion to host cells and ScaC-specific antibody is found in scrub typhus patients, immunization with ScaC was not protective. In a subsequent study, mice immunized with a zinc oxide nanoparticle-zinc oxide binding peptide-ScaA complex or an alum-zinc binding particle-ScaA complex were protected against 100 LD50 of the homologous Boryong strain^52^. ScaA is an excellent candidate for inclusion in a multiplex subunit vaccine against scrub typhus.

## The way forward

An effective vaccine to prevent scrub typhus should contain antigens of *O. tsutsugamushi* that stimulate both humoral and T cell-mediated protection. A multiplex subunit vaccine that stimulates strong humoral and T cell-mediated protection offers an approach that can be developed and tested. The strong homologous protection provided by immunization with p56 should be dissected to determine whether one or more of the hypervariable regions stimulates immunity. The strains of *O. tsutsugamushi* that are most important clinically and most prevalent epidemiologically must be identified. If the hypothesis is that one or more identified hypervariable regions stimulates immunity, then these antigenic regions from strains of public health importance can be combined in a multiplex vaccine in a platform such as recombinant adenovirus or messenger RNA. As many as 40 hypervariable regions of different strains can be expressed in a recombinant adenovirus. It should be determined whether ScaA, p47, and conserved p56 regions add to the protective immunity and merit inclusion as components of the multiplex vaccine.

Because immunization with one *O. tsutsugamushi* strain does not stimulate protective immunity against other strains, it is apparent that conserved immunodominant antigens that were present in the microorganisms in the primary infection would not be effective vaccine components. However, the identification of conserved subdominant antigens should be investigated for stimulating protective immunity by applying proven bioinformatics programs for identifying epitopes that are strongly predicted to stimulate immune protection. These predicted protective epitopes can be evaluated for stimulation of protection in animal models in pools and narrowed down to the individual protective epitopes. To determine the mechanisms and correlates of protection, experiments in valid animal models utilizing the adoptive transfer of immune serum and T cell subsets from animals immunized with the subunit antigens can be performed. In our opinion, a protective vaccine will stimulate neutralizing antibodies and cytotoxic T cells and may in the end not be overly complex or difficult to produce.

## Conclusion

Another critical challenge to the development of a useful vaccine against scrub typhus is the relatively short persistence of antibodies to *O. tsutsugamushi* and the waning of protective immunity. Fifty percent of patients who have recovered from scrub typhus revert to seronegativity at 49 weeks after infection and are susceptible to reinfection with heterologous strains of *O. tsutsugamushi* within a few months after recovery from illness^54,55^. It is possible that the interaction of the microorganism with the immune system disrupts the establishment of immune memory^56^. The failure of natural infection to stimulate durable protection suggests that a live bacterial vaccine may also fail. If this consideration is valid, the development of a multiplex subunit vaccine that employs a vaccine platform and adjuvants that trigger B cell and T cell memory would be a potentially successful approach to the important goal of developing a vaccine against the clinically and epidemiologically important strains of *O. tsutsugamushi*. A reasonable hypothesis is that a multiplex vaccine containing the conformational, neutralizing antibody-stimulating p56 hypervariable antigens of the clinically and epidemiologically most important *O. tsutsugamushi* strains and conserved antigens of p56, ScaA, and possibly p47 would be a logical approach to the unmet need of vaccine development for scrub typhus.

## Addendum

Since the final submission of this article, a valuable article has been published that includes a valid assay for neutralizing antibodies to *O. tsutsugamushi*^57^.
